# Spousal diabetes as a diabetes risk factor: A systematic review and meta-analysis

**DOI:** 10.1186/1741-7015-12-12

**Published:** 2014-01-24

**Authors:** Aaron Leong, Elham Rahme, Kaberi Dasgupta

**Affiliations:** 1 Research Institute of the McGill University Health Centre, Montreal, Quebec, Canada; 2 Department of Medicine, McGill University, Montreal, Quebec, Canada; 3 Division of Clinical Epidemiology, McGill University Health Centre, Division of Clinical Epidemiology, 687 Pine Avenue West, V Building, Montreal, Quebec H3A 1A1, Canada

**Keywords:** Diabetes mellitus, Prediabetes, Spouses concordance, Risk factor, Systematic review, Meta-analysis

## Abstract

**Background:**

Diabetes history in biologically-related individuals increases diabetes risk. We assessed diabetes concordance in spouses (that is, biologically unrelated family members) to gauge the importance of socioenvironmental factors.

**Methods:**

We selected cross-sectional, case–control and cohort studies examining spousal association for diabetes and/or prediabetes (impaired fasting glucose or impaired glucose tolerance), indexed in Medline, Embase or Scopus (1 January 1997 to 28 February 2013). Effect estimates (that is, odds ratios, incidence rate ratios, and so on) with body mass index (BMI) adjustment were pooled separately from those without BMI adjustment (random effects models) to distinguish BMI-dependent and independent concordance.

**Results:**

Searches yielded 2,705 articles; six were retained (n = 75,498 couples) for systematic review and five for meta-analysis. Concordance was lowest in a study that relied on women’s reports of diabetes in themselves and their spouses (effect estimate 1.1, 95% CI 1.0 to 1.30) and highest in a study with systematic assessment of glucose tolerance (2.11, 95% CI 1.74 to 5.10). The random-effects pooled estimate adjusted for age and other covariates but not BMI was 1.26 (95% CI 1.08 to 1.45). The estimate with BMI adjustment was lower (1.18, 95% CI 0.97 to 1.40). Two studies assessing between-spouse associations of diabetes/prediabetes determined by glucose testing reported high concordance (OR 1.92, 95% CI 1.55 to 2.37 without BMI adjustment; 2.32, 95% CI 1.87 to 3.98 with BMI adjustment). Two studies did not distinguish type 1 and type 2 diabetes. However given that around 95% of adults is type 2, this is unlikely to have influenced the results.

**Conclusions:**

Our pooled estimate suggests that a spousal history of diabetes is associated with a 26% diabetes risk increase. Recognizing shared risk between spouses may improve diabetes detection and motivate couples to increase collaborative efforts to optimize eating and physical activity habits.

## Background

The diabetes epidemic represents an escalating challenge worldwide [1], placing substantial strains on health care systems in terms of morbidity, mortality and cost associated with managing the disease and its complications [2]. Moreover, 30% to 40% of diabetes cases remain undiagnosed [3-6]. Risk assessment tools (for example, Finnish Diabetes Risk Score and Canadian Diabetes Risk Questionnaire [7,8]) may facilitate identification of at-risk individuals. Early detection allows timely management to prevent diabetes-related complications.

Diabetes history in biologically-related family members is a key component of the diabetes risk assessment [7,8]. Risk increases twofold with diabetes in one parent and fivefold with diabetes in both parents. Sibling history almost triples diabetes risk [9]. From familial aggregation studies, the heritability of type 2 diabetes is estimated at approximately 25% [10,11]. Thus far, more than 60 common genetic variants implicated in the disease have been identified through genome-wide association studies. However, their added effects explain less than 10% of the heritability of type 2 diabetes [12-14].

While heritable factors are important, socio-environmental influences are critical for the expression of genetic risk. The 21^st^ century socio-environment appears to be optimal for such expression. There has been a shift in food consumption from home-prepared regular meals to erratic and purchased meals that are energy-dense, ‘supersized’ and aggressively marketed [15,16]. The advent of modern technologies has led to reliance on internet transactions, smart phone communications and social networking, resulting in lower labor and transportation-related physical activity [15-17]. Nonetheless, despite the broad reach of these socio-environmental influences, their impact may differ from person to person and potentially from household to household.

Within households, in addition to ‘biological’ clustering of disease (that is, genetic), there may be ‘social’ clustering. This may be captured by estimating spousal concordance. Spouses are generally genetically unrelated but may share common living environments, resources, social habits, eating patterns, physical activity levels and other health behaviors [18-21]. This may be through the emergence of shared habits after marriage or behavioral similarities at the outset as a result of non-random or assortative mating.

We evaluated spousal diabetes concordance through systematic review and meta-analysis. One study published in 2009 [22] examined spousal concordance of several major coronary risk factors but performed a less comprehensive search and had limited focus on diabetes, identifying fewer studies than we present herein. The importance of more carefully and specifically estimating shared diabetes risk within couples lies in the potential for more effective screening strategies and better prevention and management that could stem from greater collaborative effort between partners to achieve changes in health behaviors [23].

## Methods

### Data sources and searches

We conducted our systematic review in accordance with Meta-analysis of Observational Studies in Epidemiology (MOOSE) standards (Additional file 1) [24]. Three citation indices, Medline, Embase and Scopus, were searched using an OVID platform. The search string was developed to identify observational studies that addressed the following: ‘Are spouses of individuals with diabetes more at risk of diabetes than spouses of individuals without diabetes?’ Subject headings and keywords included ‘diabetes’ or ‘Diabetes Mellitus’ and ‘spouse’, ‘wife’, ‘husband’, ‘couple’, ‘married’ or ‘partner’ and ‘concordance’, ‘similar’, ‘correlation’ or ‘parallel’ (Additional file 2). The search strategy was limited to articles published between 1 January 1997 and 28 February 2013, arguably an era of ‘modern’ diabetes care. The language of publication was not restricted. We manually examined reference lists of retrieved studies to identify additional potentially relevant publications (that is, citation tracking). When articles included overlapping data, only the most comprehensive was retained.

### Study selection

Each abstract was appraised independently (KD, investigator, and SP, research assistant) for relevance. Differences in opinion were resolved by consensus and/or discussion with a third reviewer (AL, investigator). We used the following inclusion criteria: 1) cross-sectional, case–control or cohort design; 2) study population with married couples selected from public health records, or administrative, hospital or clinic databases; 3) outcomes were diabetes and/or prediabetes, defined as impaired fasting glucose (IFG) or impaired glucose tolerance (IGT) [25]; and 4) effect measures reported as adjusted or unadjusted odds ratios (OR), risk ratios, hazard ratios or rate ratios. We excluded studies that did not specifically address spousal concordance and those that reported simple linear correlations of metabolic syndrome criteria only. We excluded studies that examined between-spouse correlations for absolute glucose levels rather than diabetes given that there is high intra-individual variability within both the abnormal and normal absolute glucose range [26,27]. In contrast, a diabetes diagnosis generally requires a clinical assessment that includes more than one glucose measurement and/or glycated hemoglobin or glucose tolerance testing [7,28].

### Data extraction and quality assessment

Study data were independently abstracted by two investigators (AL and KD) using standardized forms (first author, year of publication, source population, country, study design, funding sources, age restriction, proportion of spouses with diabetes (exposure), prevalence and/or incidence of diabetes in the other spouse (outcome), duration of exposure, and effect measures with 95% confidence intervals and variables used for adjustment, such as age, body mass index (BMI), marriage duration, socioeconomic status (SES)). We also abstracted information related to prediabetes, when reported. Where appropriate, we contacted authors to provide us with additional data.

The abstracts and method sections of non-English articles were translated with the assistance of native speakers of the respective languages and online translation tools. Study quality was evaluated using a modified Newcastle-Ottawa Assessment Scale for Nonrandomized Studies [29] that considered the following three potential biases: 1) selection (Were the exposed group and non-exposed group drawn from the same representative samples?); 2) comparability (Were the exposed and non-exposed groups comparable?); and 3) misclassification (Was the method of ascertainment of exposure status ‘gold-standard’ and similar between cases and non-cases?). For comparability scores, we assigned one star (*) if reported estimates were adjusted for age and another star (*) if estimates were adjusted for SES measures. Age was selected as the most important variable given that diabetes risk increases with age [30]. SES was chosen as the second important variable as SES in both married partners could explain some of the shared diabetes risk [31,32]. We adapted the scoring system for two questions on the assessment scale for purposes of this study. We awarded one additional star if blood glucose testing was used to ascertain exposure under the section ‘Selection’ for question number 3 (that is, ascertainment of exposure, Additional file 3). Similarly, we awarded an additional star if blood glucose testing was used to assess outcome, under the section ‘Outcome’ for question 1 (that is, ascertainment of outcome, Additional file 3).

### Data synthesis and analysis

All data analyses were performed using STATA (version 11 StataCorp, College Station, TX, USA). We extracted the reported effect estimates (that is, ORs, incidence rate ratios, and so on) and 95% confidence intervals from each study to generate forest plots and visually inspected for heterogeneity across studies. We were interested in comparing effect estimates in models that did not adjust for BMI (that is, considered BMI to be along the causal pathway) with those that did, in order to capture associations likely mediated directly through physical activity and dietary patterns independent of BMI. Therefore, we generated forest plots and meta-analyzed effect estimates that adjusted for possible confounders (for example, age and/or SES) but not BMI separately from models that additionally included BMI.

The single longitudinal cohort study [9] that we identified was meta-analyzed with the cross-sectional prevalence studies under the following assumptions: diabetes incidence is low (<10%) and unchanging over the time period considered, study populations are in a steady-state, and the average duration of diabetes is the same for those exposed and unexposed (that is, exposure status does not influence duration). When these conditions are met, the prevalence OR approximates incidence rate ratios [33]. In sensitivity analyses, we excluded the longitudinal study to assess for any changes in the pooled estimate.

We used DerSimonian & Laird random-effects models which account for both within-study and between-study variability to estimate the pooled effect measures with 95% confidence intervals and calculated the Higgin’s I-squared statistic that provided a percentage of variance between studies attributable to chance. I-squared estimates ≥50% were interpreted as evidence of high heterogeneity [34].

## Results

**Figure 1:**
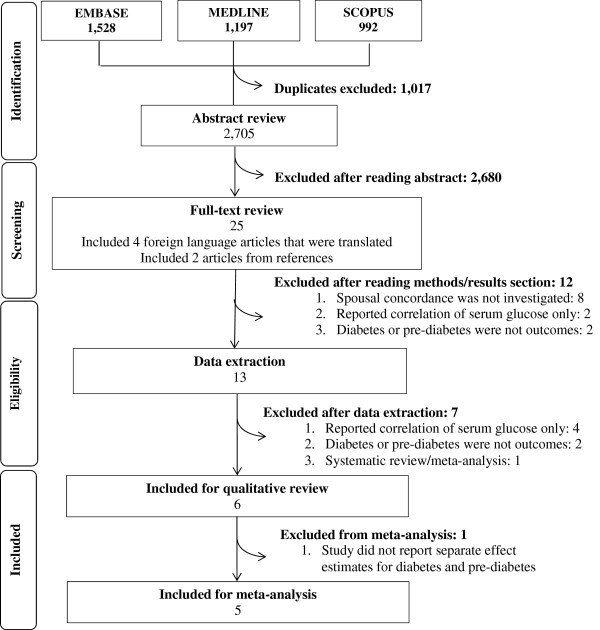
Selection strategy.

The search strategy identified 2,705 unique abstracts. Among these, 25 were determined to be potentially relevant for full text review (Figure 1), of which two were from citation tracking of review articles, and four were published in a language other than English (one Spanish, one Portuguese, one Czech and one Russian). Following full text review, 19 were removed for the following reasons: 1) did not investigate spousal concordance; 2) diabetes and/or prediabetes were not outcomes; or 3) only linear between-spouse correlations in glucose levels were investigated. One additional study was a systematic review and meta-analysis by Di Castelnuovo and colleagues [22] that pooled three studies on spousal concordance of diabetes among other major coronary risk factors; these three studies [20,35,36] were also identified through our search strategy and included in the systematic review herein. Ultimately, six studies fulfilled eligibility criteria.

The six observational studies included were conducted in different parts of the world (Table 1). Two focused on East Asian populations (China [20]; Korea [37]); two were from the United Kingdom [36,38] and included an ethnocultural mix in which more than half were Europid and the remainder were of South Asian, East Asian or African origin; one study examined naturalized Hispanics in the United States (US) [35]; and one study was from Sweden (northern European population) [9].

**Table 1:** Study characteristics and spousal association effect estimates

**Author, Year**	**Study design, time frame**	**City, Country**	**Age limits; mean age (years; SD)**	**Source population; number**	**Couples, number**	**Method of identifying diabetes**	**Diabetes prevalence**^**a**^**; number (%)**		**Marriage duration, years**	**Effect estimates (Odds ratio (95% CI) unless otherwise stated)**		**Other variables used to adjust effect estimates**
							**Husbands**	**Wives**		**Not adjusted for BMI**	**Adjusted for BMI**	
Stimpson, 2005	Cross- sectional; 1993-1994	Southwest USA	≥65;73.9 (6.3) for men; 70.9 (5.2) for women	Hispanic established populations for the epidemiologic studies of the elderly; 3,050	503	Self-report	25 (4.5)	24 (4.3)	Unclear	1.64 (1.07-2.54) women as outcome; 1.77 (1.14-2.74) men as outcome	1.53 (0.98-2.39) women as outcome; 1.78 (1.14-2.79) men as outcome	Men’s age, education, nativity, blood pressure, smoking status and alcohol intake
Jurj, 2006	Cross-sectional; 1997-2000	Shanghai, China	40-70; 54.6 (9.7) for men;51.9 (8.8) for women	Shanghai Women’s health Study between; 74,943 women	66,130	Self-report	2,689 (4.5); age-adjusted	2,469 (3.4); age-adjusted	Median 23.1	1.1 (1.0-1.3) women as outcome	1.1 (1.0-1.3) women as outcome^c^	Women’s age, education, occupation and family income
Hippisley-Cox, 2002	Cross-sectional^b^	Trent, UK	30-70	Trent Focus Collaborative Research Practice Network; 29,014	8,386	Electronic medical records; code for diabetes or current prescription of anti-hyperglycemic agents	300 (3.6)	156 (1.9)	Unclear	1.70 (1.06, 2.74) women as outcome	1.41 (0.87, 2.26) women as outcome	Women and men’s age, smoking status, GP practice clustering
Hemminki, 2010	Longitudinal cohort; 1972–2007; Mean follow-up 14.8 years	Sweden	>39	Multigeneration and hospital discharge registers, 157,549	3,490,178 person-years	Hospital discharge summariesdiagnoses	3,286	3,178	Unclear	SIR 1.31 (1.26-1.35) men as outcome; 1.33 (1.29-1.38) women as outcome	N/A	Standardized to expected number of cases for age, sex, period, region and SES
Khan, 2003	Cross-sectional^b^	London, UK	N/A; 57.4 (8.2) spouses of controls; 57.1 (7.2) spouses of participants with diabetes	Inner London GP diabetes clinic; 479 patients with diabetes for ≥5 years	245 spouses of participants with diabetes; 234 spouses of controls	WHO criteria for diabetes diagnosis	19 (7.8) spouses of diabetes patients; 7 (3.0) spouses of controls had diabetes.		Unclear	N/A	2.11 (1.74-5.1)	None
						**Method of identifying pre-diabetes/diabetes**	**Pre-diabetes/diabetes prevalence**^**a**^**; number (%)**					
Khan, 2003	See above	See above	See above	See above	See above	WHO criteria for diabetes, IGT and IFG diagnosis	28 (11.4) spouses of diabetes patients; 15 (6.4) spouses of controls		Unclear	N/A	2.32 (1.87, 3.98)	None
Kim, 2006	Cross-sectional; 1998-2001	Korea	≥10; 47.9 (12.8)	Korean National Health and Nutrition Examination Surveys; 19,541	3,141	FPG ≥6 mmol/L oranti-hyperglycemic medication	530 (16.9)		Unclear	N/A	1.92 (1.55, 2.37) women as outcome*; 1.94 (1.57, 2.40) men as outcome*	N/A

^**a**^Studies that examined only diabetes are reported in the upper half of the table; studies that examined both pre-diabetes and diabetes are reported in the lower half of the table^b^ year of data collection was not explicitly stated in published study. ^**c**^Authors reported that adjustment for BMI did not change estimates by more than 10%. | two readings of FPG ≥7 or random glucose ≥11.1 mmol/L was the criteria used to diagnose diabetes; FPG 6.0 to 6.9 mmol to diagnose IFG; OGTT 7.8 to 11.0 to diagnose IGT. FPG, fasting plasma glucose; IFG, impaired fasting glucose; IGT, impaired glucose tolerance; GP, general practitioner; OGTT: oral glucose tolerance test; SES: socioeconomic status; SIR: standardized incidence ratio.

### Quality assessment

Two key study strengths were identified. The first was performing systematic glucose testing on all participants as it ensured that all spouses had an equal opportunity to be detected to have diabetes. All participants underwent oral glucose tolerance testing in the study by Khan and colleagues [38] while, in the study from Kim and colleagues [37], fasting glucose measurements were used to detect diabetes. The second important study strength was the ability to capture diabetes incidence over time. The longitudinal cohort study by Hemminki and colleagues [9] followed 157, 549 subjects for an average of 14.8 years and was thus able to assess the impact of spousal diabetes on incident diabetes (Additional file 4).

Methods of diabetes ascertainment differed across studies (Table 1). Two evaluated a combined outcome that included prediabetes in addition to diabetes [37,38]. Khan and colleagues [38] (United Kingdom, UK) performed oral glucose tolerance testing in all couples, and diabetes was distinguished from prediabetes. Stimpson and colleagues’ study [35] (Hispanic Americans) relied exclusively on self-report for diabetes. Jurj and colleagues [20] (Shanghai, China) used self-reported diabetes for wives and wife-reported diabetes for husbands. Two studies employed health administrative database diabetes definitions. Specifically, in the UK evaluation by Hippisley-Cox and colleagues [36], diabetes status was determined through a read code for diabetes or a current prescription for anti-hyperglycemic agents or insulin from electronic medical records. In the Swedish study by Hemminki and colleagues [9], the study population was defined through the Swedish Multigenerational Register; classification as diabetes in this study required a hospital discharge diagnosis of diabetes (Hospital Discharge Register) and thus did not capture non-hospitalized cases treated only in an outpatient setting.

The sixth study, a cross-sectional analysis from Korea by Kim and colleagues [37], performed fasting glucose measurements. The outcome included a value ≥6 mmol/L or self-reported use of anti-hyperglycemic medication or self-reported diabetes; diabetes was thus combined with prediabetes. The UK study by Khan and colleagues [38] permitted not only evaluation of diabetes alone but also a combined outcome with prediabetes.

In terms of sampling strategies and source populations, the Korean National Health and Nutrition Examination Survey evaluation employed a general population-based sampling strategy [37]. The Swedish study sampled individuals in a population registry but restricted analyses to those ≥39 years of age [9]. The Shanghai study focused on women with some queries on the spouses’ health status [20]. Stimpson and colleagues examined an older Hispanic origin population (age ≥65 years) [35]. The remaining two studies examined patients registered in general practice networks [36,38]. With the exception of the Korean study by Kim and colleagues [37], studies restricted their investigation to adults ≥30 years old as older participants would be more likely to have type 2 instead of type 1 diabetes.

Two studies documented shared health-related behaviors (that is, dietary intake) within couples [20,37]. Half of the studies included a measure of SES in adjustments [9,20,35]. The Shanghai-based study [20] stratified analyses by length of co-habitation (< versus ≥23 years; median); this did not change effect estimates. In the UK study by Khan and colleagues [38], all couples were married for at least 5 years.

### Results of individual studies

Effect estimates for associations of spousal diabetes history with prevalent diabetes, adjusted for age and other covariates but not BMI, varied from as low as 10% (OR 1.1 (95% CI 1.0 to 1.3)) in the Shanghai-based study by Jurj and colleagues [20] to approximately 70% (OR 1.70 (95% CI 1.06 to 2.74)) in the Trent UK general practice study by Hippisley-Cox and colleagues [36] and in the American Southwest study on a Hispanic population by Stimpson and colleagues [35] (OR 1.64 (95% CI 1.07 to 2.54) diabetes in wives as outcome; OR 1.77 (95% CI 1.14 to 2.74) diabetes in husbands as outcome). Intermediate between these values was the Swedish cohort study by Hemminki and colleagues [9] (standardized incidence ratios 1.31 (95% CI 1.26 to 1.35) for men; 1.33 (95% CI 1.29 to 1.38) for women). While effect estimates that did not adjust for BMI were generally stronger than more fully-adjusted associations, the largest effect size was approximately a doubling of diabetes risk reported in the UK study by Khan and colleagues [38], wherein estimates were in fact adjusted for both age and BMI (OR 2.11 (95% CI 1.74 to 5.1)); it is possible that the estimate would have been even higher without BMI adjustment.

Two studies evaluated spousal associations for the combined outcome prediabetes/diabetes [37,38]. The Korean study by Kim and colleagues reported an OR for prediabetes/diabetes of 1.92 (95% CI 1.55 to 2.37) in women and 1.94 (95% CI 1.57 to 2.40) in men after adjusting for age; no BMI-adjusted associations were reported. The UK study by Khan and colleagues demonstrated that the risk of prediabetes/diabetes was also more than twofold for those with a spouse with prediabetes/diabetes (OR 2.32 (95% CI 1.87 to 3.98); adjusted for age and BMI).

### Meta-analyses

**Figure 2:**
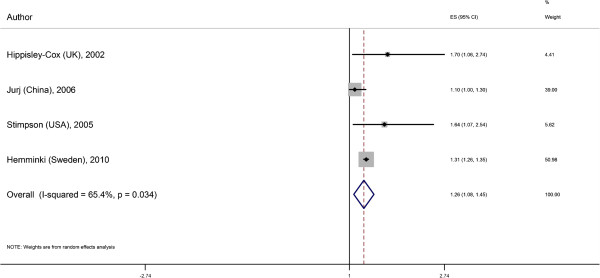
**Spousal association for diabetes not adjusted for BMI.** ES: effect size; CI: confidence interval; Hippisley-Cox (UK) reported ORs for diabetes adjusted for age; Jurj (China) adjusted for women’s age, education, occupation and family income; Stimpson (US) adjusted for age, education and nativity of husband; Hemminki (Sweden) reported rate ratios standardized to expected number of cases for age, sex, period, region and socioeconomic status; Khan (UK) reported BMI-adjusted estimates only and was therefore not pooled in this analysis. When the sexes were analyzed separately, we arbitrarily chose to display the effect estimates with diabetes in the husband as the exposure and diabetes in the wife as the outcome. In general, the effect sizes were similar whether women or men were the exposure. BMI, body mass index; OR, odds ratio.

**Figure 3:**
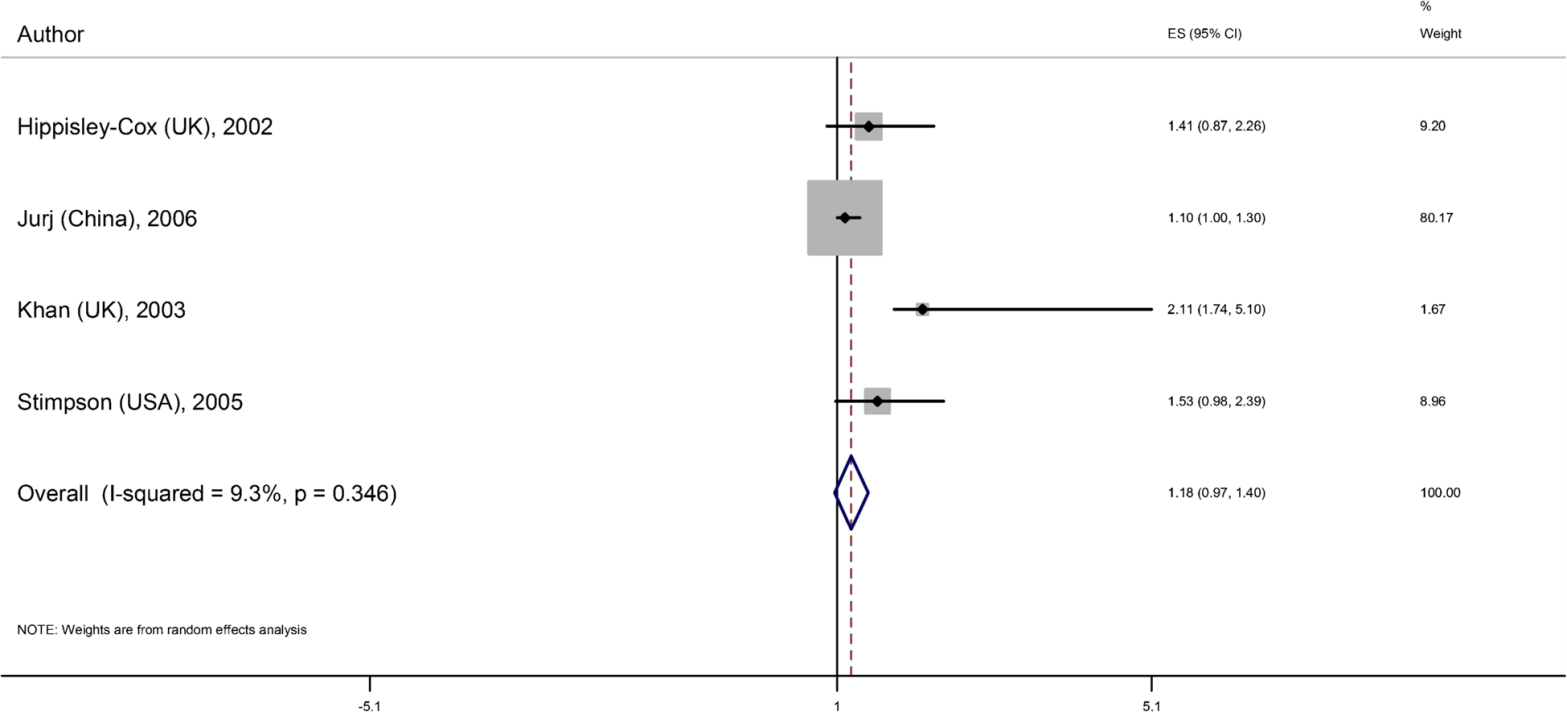
**Spousal association for diabetes adjusted for BMI**. ES, effect size; CI, confidence interval; In addition to adjusting for BMI, Hippisley-Cox (UK) reported odds ratios for diabetes adjusted for women and men’s age, smoking status, general practice clustering; Jurj (China) adjusted for women’s age, education, occupation and family income; Khan (UK) adjusted for age; Stimpson (US) adjusted for age, education, nativity, blood pressure, smoking status and alcohol intake of the husband. Hemminki (Sweden) did not report BMI-adjusted effect estimates and was, therefore, not pooled in this analysis. When the sexes were analyzed separately, we arbitrarily chose to display the effect measures with diabetes in the husband as the exposure and diabetes in the wife as the outcome. In general, the effect sizes were similar whether women or men were the exposure (Table 1). BMI, body mass index.

We excluded Kim and colleagues’ study from the meta-analysis as separate outcomes for prediabetes and diabetes were not reported. The remaining five studies evaluated a total of 75,498 couples with mean ages of 52 to 74 years. Studies ranged in sample size from 503 to 66,130 couples. By random-effects analyses, the overall effect estimate for diabetes in those with a spousal diabetes history was 1.26 (95% CI 1.08 to 1.45; adjusted for age and/or other covariates but not BMI; Figure 2). There was some evidence of heterogeneity (Higgin’s I-squared statistic = 65.4%, *P*-value = 0.03). The pooled adjusted association adjusted for BMI in addition to other covariates was 1.18 (95% CI 0.97 to 1.40; Figure 3) with less suggestion of heterogeneity (I-squared statistic = 9.3%, *P*-value = 0.35).

Given that the longitudinal cohort study by Hemminki and colleagues reported rate ratios that may differ from prevalence ORs, we separately pooled the remaining cross-sectional studies; this yielded a similar overall OR (1.33 (95% CI 0.90 to 1.76, I-squared statistic = 46.8%, *P*-value = 0.16)) although the 95% CI was wider.

## Discussion

Our analyses demonstrate spousal diabetes concordance. The degree of concordance estimated was lowest in a study that relied on women’s reports of diabetes in themselves and their spouses (effect estimate 1.1, 95% CI 1.0 to 1.30) [20] and highest in a study with systematic assessment of glucose tolerance (2.11, 95% CI 1.74 to 5.10) [38]. The random effects pooled estimate suggests that a spousal history of diabetes is associated with a 26% risk increase for diabetes overall without adjustments for BMI (effect estimate 1.26, 95% CI 1.08 to 1.45) and 18% with BMI adjustment (effect estimate 1.18, 95% CI 0.97 to 1.40). This effect size is similar to the incidence risk increase of approximately 30% attributed to spousal diabetes that is reported by the single longitudinal cohort study (standardized incidence ratios 1.31 (95% CI 1.26 to 1.35) for men; 1.33 (95% CI 1.29 to 1.38) for women) [9].

The between-spouse association was higher for the broader definition of ‘dysglycemia’ that encompassed prediabetes (IGT, IFG) and diabetes in the two studies that examined this issue, with an approximately two-fold risk increase for dysglycemia with spousal dysglycemia history (OR 1.92, 95% CI 1.55 to 2.37 by Kim and colleagues [37]; OR 2.32, 95% CI 1.87 to 3.98 by Khan and colleagues [38]). This broader definition potentially improves the power to detect spousal associations. Prediabetes, the early stage of abnormal glucose handling, is associated not only with a marked risk increase for the development of diabetes but also with an elevated risk of fatal cardiovascular outcomes and all-cause mortality [39,40].

There was some heterogeneity across the studies examined, likely partly resulting from differences in diabetes/prediabetes ascertainment methods and also perhaps to study population differences in ethnocultural composition. Differences in diabetes risk across ethnocultural groups are well-established [1,25,41,42]. Spousal diabetes history appears to increase diabetes risk both in ethnoculturally homogenous groups (for example, Hispanic, Korean and Swedish) and more diverse populations (for example, UK). The magnitude of concordance, however, differed. Notably, the Shanghai study by Jurj and colleagues demonstrated the lowest degree of shared couple risk (adjusted OR 1.1 (95% CI 1.0 to 1.3))[20]. While this may partly have resulted from misclassification of diabetes status (diabetes was self-reported for wives and wife-reported for husbands), we speculate that a delay in adopting a ‘western’ obesogenic lifestyle in China may have contributed to the lower between-spouse association detected.

Obesity has been demonstrated to spread within social networks [43] wherein norms are often shared. Our meta-analyses demonstrate that diabetes, an obesity-related complication, is also frequently concordant within a social relationship, that between spouses. As expected, spousal concordance for diabetes alone and prediabetes/diabetes were somewhat attenuated with adjustments for BMI. Interestingly, however, the signal for concordance remained even after adjustments that included BMI, suggesting that high BMI alone does not fully explain shared diabetes risk. In two of three studies that provided estimates with and without BMI adjustment, including BMI in the model did not alter the associations [20,35]. Other contributory factors may include similarities in dietary composition and food environment, physical activity, cigarette smoking and alcohol consumption [18-21].

Recognizing the presence of shared diabetes risk in couples could lead to greater cooperation and collaboration towards adoption of optimal eating and physical activity patterns and behaviors [44,45]. The importance of these in reducing diabetes risk has been demonstrated in large diabetes prevention trials around the world [46-49]. Findings from our systematic review and meta-analyses may inform strategies that shift the focus from optimizing diabetes prevention efforts in the individual with diabetes alone to optimizing couple-based interventions that enhance support and collaboration between partners. Further, a home environment in which both parents opt for healthy dietary choices and seek opportunities for physical activity could result in child health benefits, in terms of prevention of overweight/obesity, diabetes and cardiovascular disease [9,50].

Spousal diabetes concordance is also a potential tool for earlier diabetes detection. The majority of diabetes patients are diagnosed and followed in a primary care setting [51]; the results of our review suggest that diabetes diagnosis in one spouse may warrant increased surveillance in the other. Men are less likely than women to undergo regular medical evaluation after childhood [52,53] and that can result in delayed diabetes detection. Thus, men with a spousal diabetes history may particularly benefit from increased surveillance.

### Strengths and limitations

We employed a broad search strategy without language restriction. Relevant citations in retrieved articles were also examined. Study selection, quality assessment and data abstraction were performed by at least two individuals. The studies were determined to be of medium to high quality and were conducted in different regions around the world involving different ethnocultural groups. Compared to the meta-analysis by Di Castelnuovo and colleagues, our diabetes-related search string (Additional file 2) was more detailed, including ‘diabetes’ and other diabetes-related search terms in addition to ‘glucose’, given our specific focus on spousal diabetes concordance. Importantly, their included studies on diabetes (n = 3) formed a subset of our meta-analysis (n = 5) and did not include the study by Khan and colleagues who performed comprehensive assessments of glucose tolerance and demonstrated the highest effect size.

We did not include unpublished studies in our analyses as these generally lack the methodological rigor of published studies [54]. Some of the heterogeneity observed in the meta-analysis could be attributed to differing ethnocultural composition of study populations, diabetes/prediabetes ascertainment methods, study design, reference groups and characteristics of participants used to adjust effect estimates. Unmeasured confounders/mediating variables such as dietary information, physical activity level, marriage duration and time of diagnosis were not uniformly obtained across all included studies. Therefore, in pooling effect estimates, we generated random-effects models that accounted for between-study and within-study variability. Given the small number of studies, we were unable to perform meta-regression or subgroup analyses to describe the effect of other study characteristics on outcome measures or statistically explore the possibility of publication bias [55]. Results from individual studies should also be interpreted with caution as differences observed may be merely chance findings [56]; for example, although studies differed in ethnocultural composition, there were not sufficient numbers of studies within individual ethnocultural groups for definitive conclusions about any ethnocultural variations in spousal concordance. Only one study [37] reported unadjusted effect measures and, therefore, meta-analyses could only be performed for confounder-adjusted estimates. Individual studies may have potential limitations that impact the accuracy of our findings. For example, determination of diabetes or prediabetes status was more rigorous in some studies than others. Only two studies performed systematic glucose testing on all participants [37,38]. Another study likely captured only more advanced diabetes cases as its diabetes definition required a hospital discharge diagnosis [9]; while the probable under detection is expected to be similar for individuals with or without a spousal diabetes history, it potentially reduces power to detect spousal associations or bias effects towards the null, although this may not have been a major concern given the large sample size.

Conversely, spouses of diabetes patients could have greater understanding of diabetes and seek medical assistance in the event of relevant symptoms. Similarly, physicians may enforce greater surveillance for these spouses; this detection bias could inflate estimates of association. Two studies that identified diabetes cases from electronic health records did not distinguish between type 1 and type 2 diabetes [9,36]. However given that around 95% of diabetes in adults is type 2, this unlikely made a difference to the results. The single longitudinal cohort study by Hemminki and colleagues [9] demonstrated an effect estimate similar to the overall effect estimate identified across the cross-sectional studies, suggesting that the influence of incidence-prevalence bias (Neyman bias) associated with not capturing undiagnosed, mild or fatal diabetes cases in cross-sectional studies may be minimal when making inferences in relation to diabetes risk [57].

Spousal history appears to be a robust signal for diabetes risk that may facilitate diabetes detection. Better understanding of underlying mechanisms of concordance could allow the development of tailored strategies to leverage shared risk to achieve health behavior change. Several studies have indicated spousal concordance with respect to BMI [58-63], consumption of fat and fiber [60] and physical activity [64,65]. Shared behaviors and risk profiles may be present already at the time of marriage, through an assortative mating process wherein individuals with similar physical (for example, body mass index), ethnocultural, social (for example, social class) and behavioral (for example, eating and physical activity behaviors) characteristics may be more likely to become partners. Additionally or alternatively, spouses may shape one another’s behaviors over time or be influenced by common external factors (for example, life events, physical environment, social network), contributing to diabetes concordance. An examination of the effects of duration of marriage on spousal diabetes concordance could provide some insight in terms of the importance of changes in health behavior that occur during marriage. However, there was little information on marriage duration in the studies examined. There is, however, evidence for spousal correlations of weight change over time [65-67]. In an analysis of 32 years of follow-up data from the Framingham cohort, Christakis and Fowler demonstrated that development of obesity in a spouse increased one’s risk of obesity by 37%, comparable to the 40% risk increase from the development of obesity in a sibling [43].

Even more compelling are so-called ‘ripple effects’ described by Gorin and colleagues where interventions delivered to one spouse are demonstrated to affect the other [68]. For example, in the Women’s Health Trial, the husbands of women in the low-fat dietary intervention arm reduced their fat intake and body weight to a greater extent than the husbands of women in the control arm [69]. In the National Institutes of Health-funded Look AHEAD trial that examined the effects of weight loss on vascular disease events in diabetes patients, approximately 25% of the spouses of participants in the intensive intervention arm lost 5% or more of baseline weight compared to less than 10% of spouses of participants in the control arm [68]. This body of evidence suggests that not only can spousal diabetes concordance be leveraged to increase detection of diabetes and related risk factors, but also that diabetes prevention strategies could capitalize on within-couple influences.

Three possible strategies to examine spousal diabetes concordance and its underlying mechanisms include a prospective cohort study with more detailed data collection complemented by qualitative assessment, analysis of historical cohort data and analysis of diabetes prevention trial follow-up data. In a prospective cohort study (that is, examination of a group of married couples over time wherein half have type 2 diabetes in one partner at baseline), married couples could undergo systematic evaluation of health behaviors (for example, dietary intake interviews, food frequency questionnaires, pedometer or accelerometer-based assessments of physical activity), anthropometric measures (weight, height, fat mass), sociodemographic profiles (ethnocultural background, immigration status, education, occupation, income), living arrangements and glucose handling (oral glucose tolerance testing) for accurate classification of diabetes and prediabetes. Periodic reassessment would allow capture of incident prediabetes and diabetes to determine the impact of factors such as marriage duration and degree and duration of shared health-related behaviors. Such a study would be strengthened by in-depth interviews or focus group discussions to ascertain participants’ perceptions of concordance and its underpinnings. One could also examine spousal diabetes concordance and its relationship to marriage duration using a historical cohort design, similar to that employed by Christakis and Fowler to assess obesity concordance with Framingham cohort data. Third, evaluations for ripple effects could be conducted among individuals and spouses involved in diabetes prevention trials, namely the Diabetes Prevention Program, the Finnish Diabetes Prevention Study, the India Diabetes Prevention program and a Japan lifestyle intervention program, wherein dietary and physical activity interventions lead to relative reductions of 28% to 67% in diabetes incidence over an average of four years [46,48,70,71]; benefits of lifestyle intervention can persist beyond ten years [72]. It is possible that spouses of those randomized to the lifestyle intervention arms in these trials experienced lower incidence rates of diabetes than spouses of control arm participants.

## Conclusions

In summary, spousal diabetes history confers an increased risk for diabetes that our pooled estimate suggests is 26%. Spousal history of diabetes/prediabetes confers an approximately two-fold risk. This is comparable to the two-fold diabetes risk associated with diabetes history in one parent. Recognizing shared couple risk may result in greater support and collaboration within the family to engage in diabetes prevention efforts. Physicians and other health care professionals may use this information to encourage couple-based interventions to adopt a balanced dietary intake that is not energy-dense, make healthier food choices, and increase physical activity levels. Diabetes screening may be warranted in the partners of individuals with diabetes, to allow for early detection and prevention of diabetes-related complications. Our study thus indicates that documentation of family history may need to be more comprehensive by including spousal history and not just that of parent–child and sibling relationships. Spousal history could be incorporated into the diabetes clinical evaluation and risk assessment tools to improve their utility for identifying undiagnosed cases and at-risk individuals as part of our concerted efforts to curb the global diabetes epidemic.

## Abbreviations

BMI: Body mass index; IFG: Impaired fasting glucose; IGT: Impaired glucose tolerance; MOOSE: Meta-analysis of observation studies in epidemiology; OR: Odds ratio; SES: Socioeconomic status.

## Competing interests

The authors declare that they have no competing interests.

## Author’ contributions

The guarantor for this article is Kaberi Dasgupta (KD). KD and AL contributed to the study conception and design. AL, ER and KD analyzed and interpreted the data. AL and KD drafted the article. ER revised it critically for intellectual content. All authors read and approved the final manuscript.

## Authors’ information

KD is Associate Professor of Medicine at McGill University and holds the Fonds de recherche Santé du Québec-Société québécoise d’hypertension artérielle-Jacques de Champlain Award. ER is Associate Professor in the Department of Medicine of McGill University and holds a Senior Investigator award from the Fonds de Recherche en santé du Québec. AL is a Canadian Diabetes Association post-doctoral research fellow.

## Supplementary Material

Additional file 1 Meta-analysis for Observational Studies in epidemiology (MOOSE) Checklist. Click here for file

Additional file 2 Search strings for three citation databases. Click here for file

Additional file 3 Modified Newcastle-Ottawa quality assessment scale for nonrandomized observational studies. Click here for file

Additional file 4 Quality assessment of six included studies using a modified Newcastle-Ottawa quality assessment scale for nonrandomized observational studies. Click here for file
