# Supplementary material for: Spousal diabetes as a diabetes risk factor: A systematic review and meta-analysis
Source: BMC Med. 2014 Jan 24;12:12. doi: 10.1186/1741-7015-12-12 (PMC3900990; doi:10.1186/1741-7015-12-12)
Supplement: Additional file 1 — Meta-analysis for Observational Studies in epidemiology (MOOSE) Checklist. [file 1741-7015-12-12-S1.docx]

**Additional file 1: Meta-analysis for Observational Studies in epidemiology (MOOSE) Checklist**

| **Criteria** | **Section in manuscript** |
| --- | --- |
| **Reporting of background should include** |  |
| Problem definition | Introduction |
| Hypothesis statement | Introduction |
| Description of study outcomes | Introduction |
| Type of exposure or intervention used | Introduction |
| Type of study designs used | Introduction |
| Study population | Introduction |
| **Reporting of search strategy should include** |  |
| Qualifications of searchers | Methods: Data sources and searches |
| Search strategy, including time period included in the synthesis and keywords | Methods: Data sources and searches |
| Databases and registries searched | Methods: Data sources and searches |
| Search software used, name and version, including special features | Methods: Data sources and searches |
| Use of hand searching | Methods: Data sources and searches |
| List of citations located and those excluded, including justifications | Methods: Study selection |
| Method of addressing articles published in languages other than English | Methods: Study selection |
| Method of handling abstracts and unpublished studies | Discussion: Strengths and limitations |
| Description of any contact with authors | Methods: Data extraction |
| **Reporting of methods should include** |  |
| Description of relevance or appropriateness of studies assembled for assessing the hypothesis to be tested | Methods: Data extraction |
| Rationale for the selection and coding of data | Methods: Data extraction |
| Assessment of confounding | Methods: Quality assessment |
| Assessment of study quality, including blinding of quality assessors; stratification or regression on possible predictors of study results | Methods: Quality assessment |
| Assessment of heterogeneity | Methods: Data synthesis and analysis |
| Description of statistical methods in sufficient detail to be replicated | Methods: Data synthesis and analysis |
| Provision of appropriate tables and graphics | Table 1 |
| **Reporting of results should include** |  |
| Graph summarizing individual study estimates and overall estimate | Figure 2 and Figure 3 |
| Table giving descriptive information for each study included | Table 1 |
| Results of sensitivity testing | Results: Meta-analysis |
| Indication of statistical uncertainty of findings | Results: Meta-analysis |
| **Reporting of discussion should include** |  |
| Quantitative assessment of bias | Results: Quality assessment |
| Justification for exclusion | Discussion: Strengths and Limitations |
| Assessment of quality of included studies | Results: Quality assessment |
| **Reporting of conclusions should include** |  |
| Consideration of alternative explanations for observed results | Discussion: Strengths and Limitations |
| Generalization of the conclusions | Discussion: Conclusions |
| Guidelines for future research | Discussion: Strengths and Limitations |
| Disclosure of funding source | Financial support |
