# Supplementary material for: Spousal diabetes as a diabetes risk factor: A systematic review and meta-analysis
Source: BMC Med. 2014 Jan 24;12:12. doi: 10.1186/1741-7015-12-12 (PMC3900990; doi:10.1186/1741-7015-12-12)
Supplement: Additional file 2 — Search strings for three citation databases. [file 1741-7015-12-12-S2.docx]

**Appendix 1: Search Strings for three citation databases**

| Database | Search String |
| --- | --- |
| Medline | (Glucose Metabolism Disorders OR Blood Glucose OR Hemoglobin A, glycosylated OR blood glucose OR blood sugar OR IGT OR IFG OR a1c OR glucose intoleran* OR fasting adj2 glucose OR glucose control OR plasma glucose OR diabet* OR health status) AND (spouses OR spouses or spouse or spousal OR Family Characteristics OR wife OR husband OR couple or couples OR married or marital or marriage OR partners or partner*) AND (concordance OR similar* OR correlation OR parallel OR cluster OR environmental factor OR environmental cause OR shared environment OR risk factors) |
| Embase | Glucose Metabolism OR glucose blood level OR blood glucose OR Hemoglobin A1c glycosylated OR blood glucose OR blood sugar OR diabet* OR IGT OR IFG OR a1c OR glucose intoleran* OR fasting glucose OR glucose control OR plasma glucose OR health status) AND (spouses OR spouses or spouse or spousal OR Family Characteristics OR wife OR husband OR couple or couples OR married or marital or marriage OR partners or partner*) AND (concordance OR similar* OR correlation OR parallel OR cluster OR environmental factor OR environmental cause OR shared environment OR risk factors ) |
| Scopus | PUBDATETXT(>February 2013) ((TITLE-ABS-KEY(diabetes mellitus OR   glucose OR diabetes OR diabetic) AND TITLE-ABS-KEY(spouse OR spouses  OR spousal OR wife OR husband OR couple* OR partner*) AND TITLE-ABS-KEY(concordance OR similar* OR correlat* OR parallel ORcluster))) |
