# Supplementary material for: Spousal diabetes as a diabetes risk factor: A systematic review and meta-analysis
Source: BMC Med. 2014 Jan 24;12:12. doi: 10.1186/1741-7015-12-12 (PMC3900990; doi:10.1186/1741-7015-12-12)
Supplement: Additional file 3 — Modified Newcastle-Ottawa quality assessment scale for nonrandomized observational studies. [file 1741-7015-12-12-S3.docx]

**Appendix 2: Modified Newcastle-Ottawa quality assessment scale for nonrandomized observational studies**


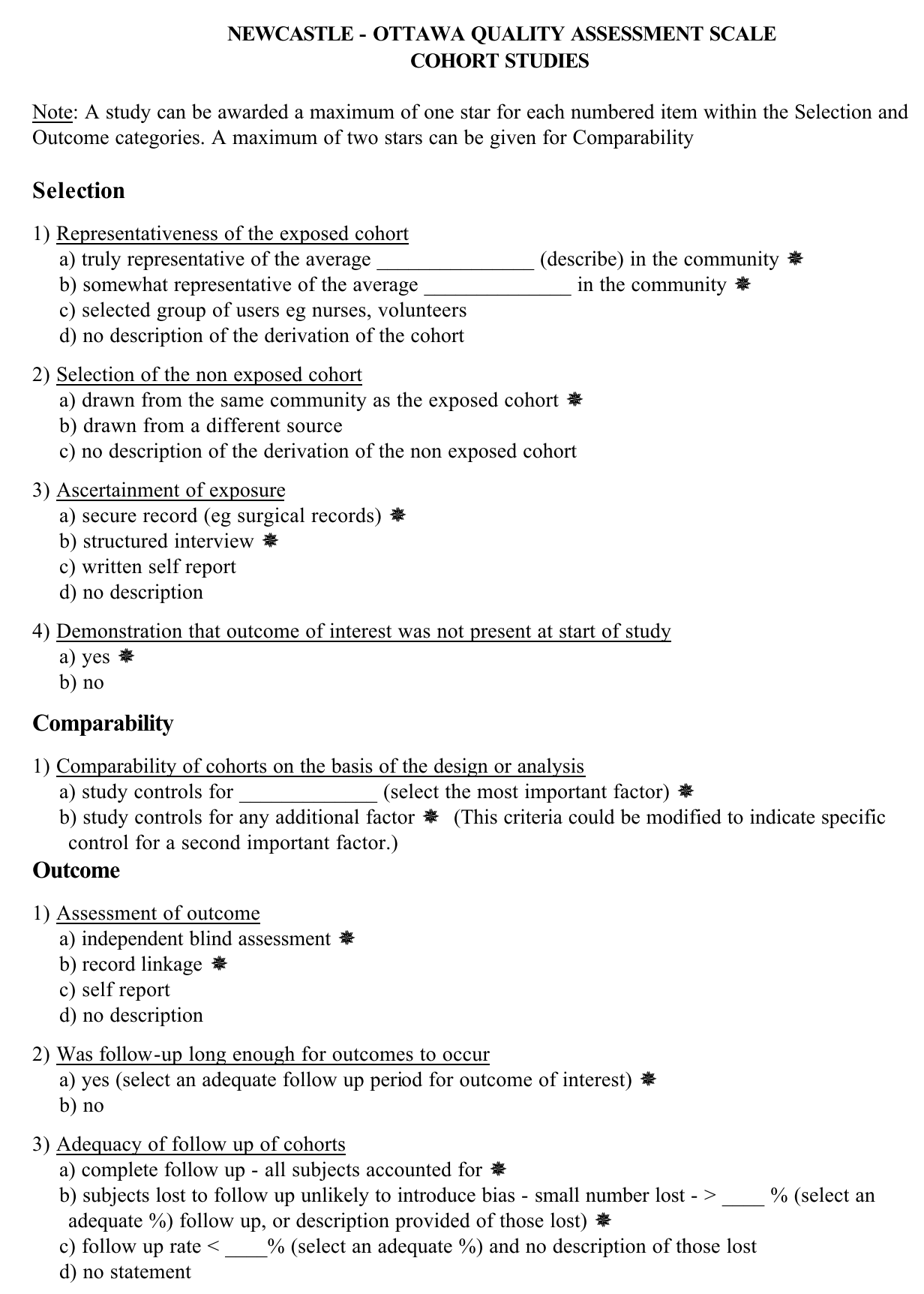


Based on the Newcastle-Ottawa scale, three domains are assessed: “selection of the study groups”, “comparability of the groups” and “ascertainment of either the exposure or outcome of interest”. For Question 3 of “Selection”, we awarded an additional star (*) if blood glucose testing was performed on all participants to ascertain exposure. For Question 1 of “Outcome”, we awarded an additional star (*) if blood glucose testing was performed on all participants to assess outcome.
