# Supplementary material for: Spousal diabetes as a diabetes risk factor: A systematic review and meta-analysis
Source: BMC Med. 2014 Jan 24;12:12. doi: 10.1186/1741-7015-12-12 (PMC3900990; doi:10.1186/1741-7015-12-12)
Supplement: Additional file 4 — Quality assessment of six included studies using a modified Newcastle-Ottawa quality assessment scale for nonrandomized observational studies. [file 1741-7015-12-12-S4.docx]

**Appendix 3: Quality Assessment of 6 included studies using a modified Newcastle-Ottawa quality assessment scale for nonrandomized observational studies**

| **Questions** | **Selection** | | | | | | | | **Comparability** | **Outcome** | | | | | |
| --- | --- | --- | --- | --- | --- | --- | --- | --- | --- | --- | --- | --- | --- | --- | --- |
|  | **1** | | **2** | | **3** | | **4** | | **1** | **1** | | **2** | | **3** | |
| Maximum no. of stars (*) | * | | * | | ** | | * | | ** | ** | | * | | * | |
| Stimpson, 2005 | a | * | a | * | b | * | b | - | ** | c | - | b | - | d | - |
|  |  |  |  |  |  |  |  |  |  |  |  |  |  |  |  |
| Jurj, 2006 | a | * | a | * | b | * | b | - | ** | c | - | b | - | d | - |
| Kim, 2006 | a | * | a | * | a | ** | b | - | * | c | - | b | - | d | - |
| Khan, 2003 | b | * | a | * | a | ** | b | - | * | a | * | b | - | d | - |
| Hippisley-Cox, 2002 | a | * | a | * | a | * | b | - | * | b | * | b | - | d | - |
| Hemminki, 2010 | c | - | a | * | a | * | a | * | ** | b | * | a | * | b | * |

Letters a through d correspond to questions responses on the quality assessment scale (Modified Newcastle-Ottawa quality assessment scale for nonrandomized observational studies). For Question 3 of “Selection”, we awarded an additional star (*) if blood glucose testing was performed on all participants to ascertain exposure. For Question 1 of “Outcome”, we awarded an additional star (*) if blood glucose testing was performed on all participants to assess outcome. For “Comparability”, the first star (*) was awarded if effect estimates were adjusted for age. The second star (*) was awarded if effect estimates were adjusted for markers of socioeconomic status.
